# *Drosophila* Prominin-like, a homolog of CD133, interacts with ND20 to maintain mitochondrial function

**DOI:** 10.1186/s13578-019-0365-0

**Published:** 2019-12-19

**Authors:** Xuexiang Wang, Huimei Zheng, Zexiao Jia, Zhaoying Lei, Mengyao Li, Qianqian Zhuang, Hui Zhou, Yue Qiu, Yong Fu, Xiaohang Yang, Yongmei Xi, Qingfeng Yan

**Affiliations:** 1 College of Life Science, Zhejiang University, Hangzhou, 310058 Zhejiang China; 2 Institute of Medical Sciences, The Second Hospital of Shandong University, Jinan, 250000 Shandong China; 3 Division of Human Reproduction and Developmental Genetics, The Women’s Hospital, Zhejiang University School of Medicine, Hangzhou, Zhejiang China; 4 Institute of Genetics, Zhejiang University, Hangzhou, 310058 Zhejiang China; 5 The Children’s Hospital, School of Medicine, Zhejiang University, Hangzhou, 310052 Zhejiang China; 6 The First Affiliated Hospital, School of Medicine, Zhejiang University, Hangzhou, 310003 Zhejiang China; 7 Key Laboratory for Cell and Gene Engineering of Zhejiang Province, Hangzhou, 310058 Zhejiang China

**Keywords:** *Drosophila* Schneider 2 cells, Prominin-like, ND20, Mitochondria, CD133

## Abstract

**Background:**

*Drosophila* Prominin-like is a homolog of mammalian CD133, which is recognized as a biomarker for stem cells. The interacting proteins of CD133 and their biological functions remain elusive.

**Results:**

In this study, we using yeast two-hybrid assays, GST pull-down assay and co-immunoprecipitation (Co-IP) methods found that *Drosophila* Prominin-like interacts with ND20, a subunit of mitochondrial respiratory complex I. Bioinformatics analysis suggests that Prominin-like is a six-transmembrane glycoprotein which localizes on cellular membranes. Immunostaining and mitochondrial fractionation indicate that *Drosophila* Prominin-like could localize in the mitochondria. The knockdown of *prominin*-*like* in S2 cells resulted in transient mitochondrial dysfunctions as evidenced by reduced ATP production, elevated ROS generation and an accompanied reduction in mitochondrial proteins. Mitochondrial dysfunctions were detected in aged *prominin*-*like* mutant flies.

**Conclusion:**

Our data indicates that Prominin-like acts to maintain mitochondrial function through its interaction with ND20 which, itself, is active in the mitochondrial electron transport chain. Our study provides insights into a novel molecular mechanism of *Drosophila prominin*-*like* and suggests a similar function of CD133 in mammals.

## Background

CD133 (AC133/prominin-1) was first identified in 1997 in human hematopoietic progenitor cells and in murine epithelia [1, 2]. It has now been widely recognized as a cell-surface marker for stem cells (CSCs) in a variety of tissues and in various cancers including breast carcinomas [3], colon cancer [4], pancreatic cancer [5], hepatocellular carcinoma [6] and neural tumors [7]. However, the exact mode of its action in stem cells or cancer cells remains controversial.

To gain insight into the biological function and the underling molecular mechanisms of CD133, various animal models such as *Drosophila* [8, 9], *Xenopus laevis* [10] and rodents [11] have been used where CD133 shows conserved cellular roles in both invertebrate and vertebrate photoreceptor cells [9]. In *Drosophila*, *prominin* and *prominin*-*like* are the only two homologs of CD133 (flybase.org). Prominin has been demonstrated to be involved in eye disc morphogenesis where Prominin selectively localizes on the stalk membrane and the tips of the microvilli that create the rhabdomere. Removal of *prominin* has been shown to disrupt the morphology and organization of the photo-transduction compartment [8]. Prominin-like has been shown to be localized to the apical protrusions of wing imaginal disc cells, but with unknown function [12]. Prominin-like might act as a protein transporter and be involved in neural development, where knock down of *prominin*-*like* in cultured primary neurons leads to neuropathological disorders [13].

Our previous study revealed that the physiological function of Prominin-like is involved in the maintenance of body size and weight in adult flies [14]. In Prominin-like mutants, the dTOR pathway and dilp6 levels were both elevated, resulting in larger body sizes and excessive weight. Loss of Prominin-like also leads to an accumulation of lipid droplets in fat body cells [15] and decreased mitochondrial β-oxidation levels in whole animal level [14]. Correspondingly, lipid droplet accumulation has recently been reported to affect mitochondrial function in *Drosophila* [16]. The *prominin*-*like* defect seemed to lead to a slowing down of energy-consuming metabolic processes. In this way, it would be interesting to elucidate the potential role of Prominin-like involved in the energy metabolism that may relate to mitochondrial function.

In the present study, we identify that ND20, a subunit of mitochondrial respiratory complex I, interacts with Prominin-like. GST pull-down and Co-Immunoprecipitation (Co-IP) experiments further confirmed that the interaction between Prominin-like and ND20 is direct. Knock down of the expression of endogenous *prominin*-*like* causes a short-term reduction of mitochondrial proteins and mitochondria dysfunction in the S2 cell line. Conditional mitochondrial dysfunction was also detected in aged *prominin*-*like* mutant flies. These findings may provide new insight towards the understanding of the molecular mechanism by which CD133 may be involved in the energy metabolism of mammalian cells.

## Results

### Structural characteristics and the cellular localization of Prominin-like

**Fig. 1:**
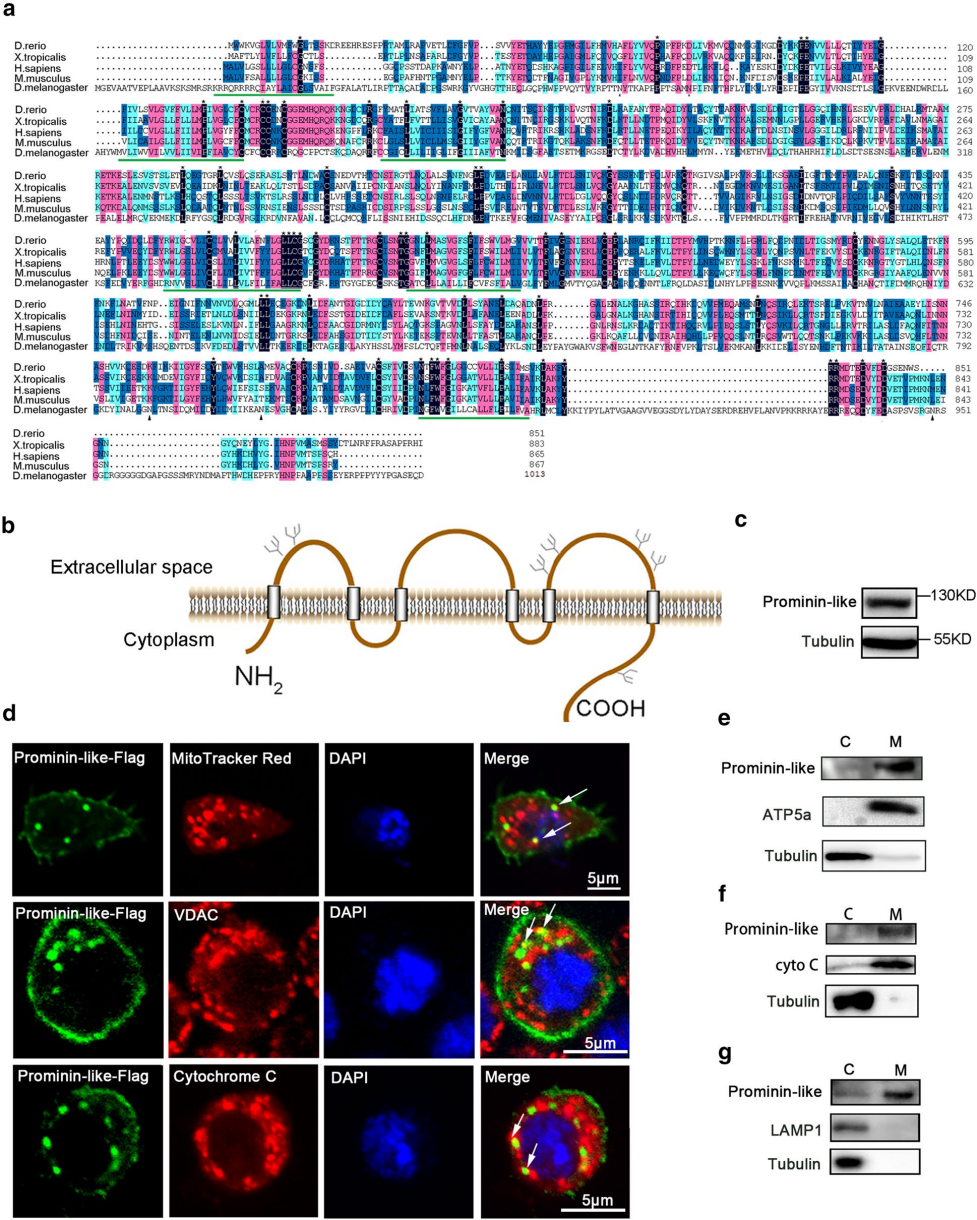
Structural characteristics and the cellular localization of Prominin-like. **a** Multiple sequence alignments of CD133 from five species; *Daniorerio, Xenopus tropicalis, Homo sapiens, Musmusculus* and Prominin-like from *Drosophila melanogaster*. Identical residues are marked with black stars, conserved residues with a blue background, and similar residues with a salmon background. Predicted transmembrane domains are underlined. Potential *N*-glycosylation sites are highlighted in triangles. **b** Membrane topology of *Drosophila* Prominin-like. Seven potential N-glycosylation sites have been drawn. **c** Western blot analysis of Prominin-like levels in untreated S2 cells. **d** S2 cells were transiently transfected with Flag-tagged Prominin-like, and colabeled with anti-Flag antibody (green), MitoTracker Red (the upper lane)/VDAC (the middle lane)/Cytochrome C (the lower lane) (red) and DAPI (blue). The merge of the green and red signals (yellow) confirms the partially mitochondrial localization of Prominin-like. **e**–**g** Western blot analysis of cytosolic and mitochondrial fractions separated by centrifugation. **e** Samples were probed with anti-Prominin-like, anti-ATP5a, and anti-tubulin antibodies. **f** Samples were probed with anti-Prominin-like, anti-Cytochrome C, and anti-tubulin antibodies. **g** Samples were probed with anti-Prominin-like, anti-LAMP1, and anti-tubulin antibodies

To evaluate the conservative properties of Prominin-like across species, we aligned the amino acid sequences of CD133 from *Homo sapiens, Mus musculus, Xenopus tropicalis, Danio rerio* with that of Prominin-like from *Drosophila melanogaster,* using open database from NCBI. Results showed that Prominin-like shares a 47.9% homology among these species (Fig. 1a). We then performed bioinformatics analysis on the structural characteristics of Prominin-like. Its hydropathic features show that Prominin-like is an integral membrane protein with six hydrophobic segments. These may constitute putative transmembrane domains and form three large extracellular loops, including two small cytoplasmic loops and an intracellular amino- and carboxyl tail (Fig. 1b). The transmembrane regions were shown to be located at 32–54, 165–187, 213–235, 492–514, 535–557 and 852–874 amino acids, respectively (Fig. 1a, underlined). Further analysis of amino acids identified seven predicted N-glycosylation sites, two in the first extracellular loop, four in the third extracellular loop and one in the intracellular carboxyl tail (Fig. 1a, triangles). This indicates that Prominin-like is a member of the six-transmembrane glycoprotein family.

To discover the cellular localization of endogenous Prominin-like, we firstly generated a polyclonal antibody against the C-terminal region of Prominin-like [14]. Although the signal of Prominin-like was not detectable by immunostaining with the antibody, Western Blot proved a clear band of endogenous Prominin-like expression (Fig. 1c). We further cloned the coding sequence of *prominin*-*like* into a flag-tagged pAC5.1 vector and transfected it into the S2 cells. Immunostaining showed that the the fusion protein, Prominin-like-flag localized on the cell membrane as well as the cytoplasm. Interestingly, the subcellular localization of Prominin-like was partially overlapped with mitochondria, as indicated by the MitoTracker Red and the endogenous mitochondria proteins, voltage-dependent anion channel (VDAC/mitochondrial porin) and Cytochrome C (Fig. 1d, arrows). The cell membrane and mitochondrial localization of Prominin-like was further demonstrated using cell fractionation experiments. Untreated S2 cells extracts were subjected to differential centrifugation, and each fraction was analyzed with Western Blot to detect the presence of Prominin-like. As shown in Fig. 1e, the Prominin-like protein was highly enriched in the fraction containing mitochondria, as determined by the mitochondrial marker ATP-synthase complex V subunit alpha (ATP5a), and it was poorly enriched in the cytosolic fraction, including the cell membrane. Similar results were found when using the mitochondrial marker Cytochrome C (Fig. 1f). To exclude the possibility that the mitochondria fraction could be contaminated with organelles in the secretary pathway, we co-stained with the lysosomal marker LAMP-1. The mitochondrial fraction was verified by the absence of LAMP-1 (Fig. 1g). These results indicate that *Drosophila* Prominin-like, as a cellular membrane protein, could localize in the mitochondria.

### Prominin-like directly interacts with mitochondrial ND20 protein

**Fig. 2:**
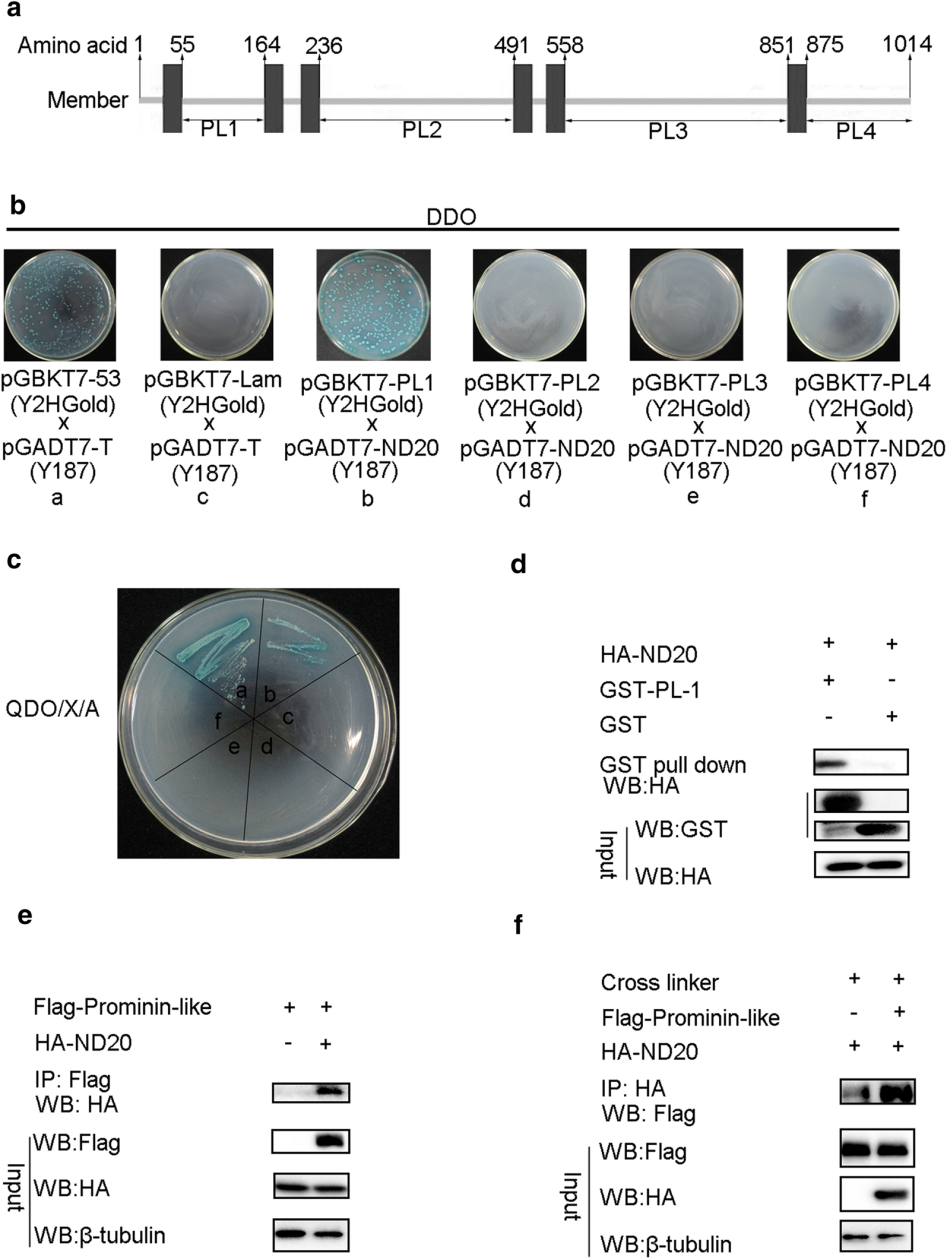
Prominin-like interacts with ND20 proteins. **a** Schematic illustration of truncated Prominin-like region. The cDNA fragments encoding amino acid: 55–164, 236–491, 558–851, and 875–1014 of *prominin*-*like* were cloned into vectors for interaction assays. **b**, **c** Yeast two-hybrid assays: pGBKT7-53 and pGADT7-T were used as positive controls with pGBKT7-Lam and pGADT7-T as negative controls; Mated yeast strains containing both bait vectors (pGBKT7-PL-1,-2,-3 or -4) and prey vectors (pGADT7-ND20) were plated on double dropout (DDO/X) (**b**) and higher stringency quadruple dropout (QDO/X/A) selective agar medium (**c**); there were one-to-one correspondences among **a**–**f** in **b** and **c**. **d** GST pull-down assays. GST or GST-PL-1 fusion protein generated by *E. Coli* BL21 cells was purified by glutathione agarose resin, followed by incubation of the resin with HA-ND20 protein expressed in S2 cells. After washing with PBS, the bound proteins were analyzed by Western blotting with anti-HA antibodies. **e**, **f** Co-IP assay analysis of the interaction between Prominin-like and ND20. Cells were co-transfected with pAC5.1-Flag-Prominin-like and pAHW-HA-ND20 in S2 cells; co-transfection of pAC5.1-Flag and pAHW-HA served as negative controls. Some of the lysates were subjected to input assays to assess β-tubulin, Flag-Prominin-like and HA-ND20 protein levels, and the remainder were subjected to IP assays. **e** HA-ND20 proteins were detected by Western blotting using anti-HA antibodies. **f** Flag-prominin-like proteins were detected by Western blotting using anti-Flag antibodies in the presence of the cross linker, DTSSP

To identify interaction proteins of Prominin-like, we focused on several putative genes including HRB27C, TSG101 (CG9712), and ND20 (CG9172), which were reported in the public data base (http://www.flybase.org). We performed yeast two-hybrid assays to screen the candidate proteins. As Prominin-like is a transmembrane glycoprotein, we cloned the four relatively larger fragments between the transmembrane domains, the three large extracellular loops (amino acid: 55–164/PL1, 236–491/PL2, 558–851/PL3), and the cytoplasmic C-terminal domain (amino acid: 875–1014/PL–4) (Fig. 2a) into the pGBKT7 plasmids as the bait, respectively. The full-length cDNA of all three candidate genes were then cloned into three respective pGADT7 vectors as prey. Interestingly, of these putative interaction proteins, only ND20, a subunit of mitochondrial respiratory complex I, showed strong interaction with PL1 as indicated by α-galactosidase activation in the presence of X-a-Gal (Fig. 2b, c). We then conducted glutathione S-transferase (GST) pull-down assays to verify this interaction. GST and GST-PL1 fusion proteins purified from bacteria were transformed with the pGEX-4T-1 or pGEX-GST-PL1 recombination plasmids, respectively, and bound to glutathione sepharose beads. The lysate from S2 cells expressing HA-ND20 was added to the mixture. The precipitates were examined by Western blotting using an anti-HA antibody. HA-ND20 was precipitated by GST-PL1, but not by GST alone (Fig. 2d). The lysates from S2 cells expressing HA-TSG101 and HA-HRB27C were also added to the bead mixture. These were not pulled down by either GST or GST-PL1 (Additional file 1: Figure S1). These results suggest that the first extracellular loop (PL1) of Prominin-like interacts with ND20 directly in vitro.

To confirm the interaction between Prominin-like and ND20 in vivo, we further performed co-immunoprecipitation (Co-IP) assays. Full-length Flag-tagged Prominin-like was co-transfected with HA-tagged ND20 in S2 cells. Following the immunoprecipitation of Flag-Prominin-like, HA-ND20 was detected in the immune complexes (Fig. 2e). In a converse co-IP experiment, Flag-Prominin-like was also recovered in the immune complexes precipitated by HA-ND20 in the presence of the cross linker (Fig. 2f). Taken together, these results demonstrate that ND20 could interact with Prominin-like and the interaction position was at the first extracellular loops of Prominin-like.

### A deficiency of Prominin-like influences cell growth and ATP production

**Fig. 3:**
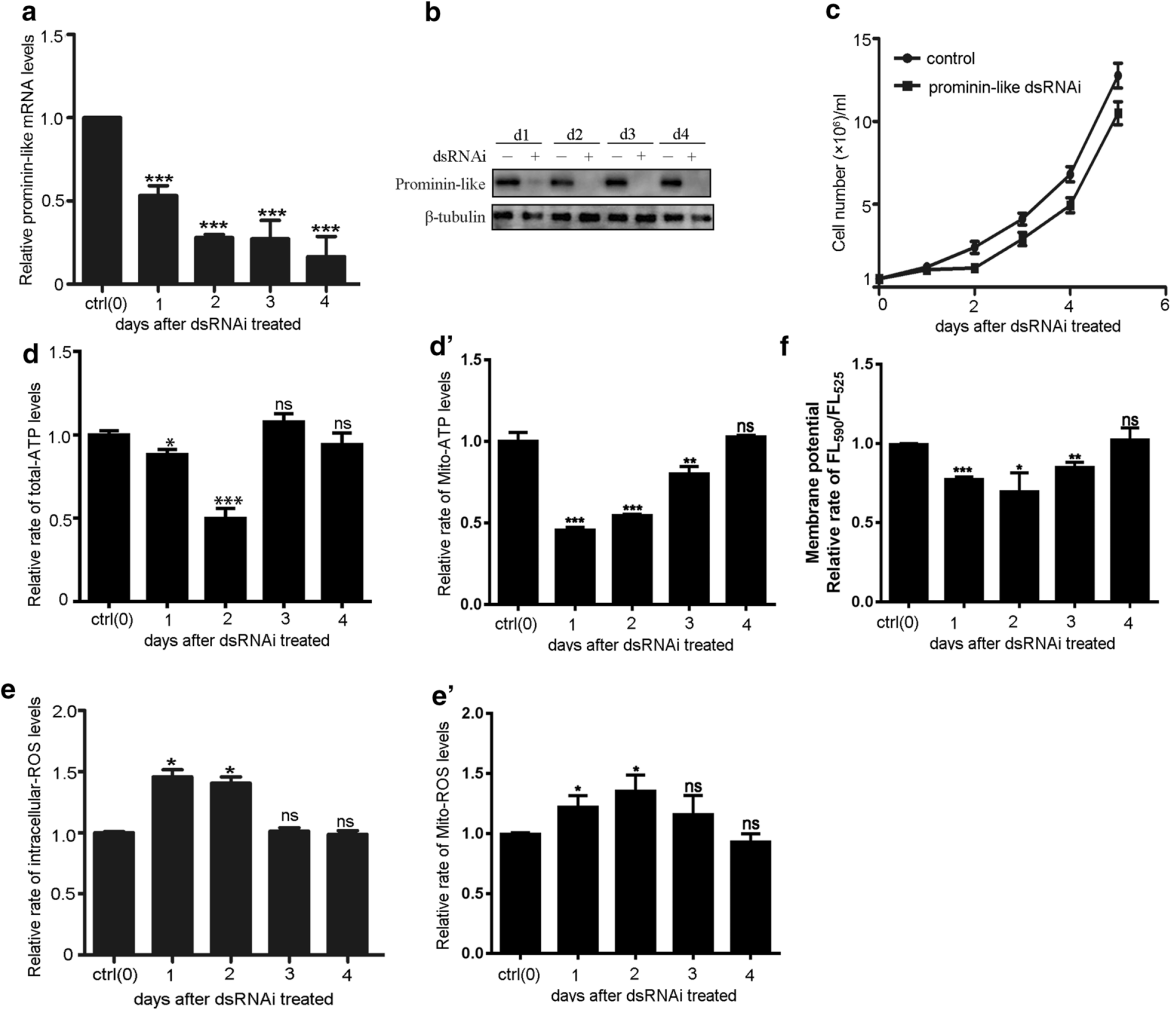
The deficiency of Prominin-like influences cell growth and ATP production. **a** Quantitative real-time PCR was used to analyze *prominin*-*like* mRNA levels and to test the efficiency of the dsRNA treatment for 4 days in S2 cells. **b** Western blot examination of Prominin-like protein levels after *prominin*-*like* dsRNAi treatment for 4 days. **c** Cell growth curve analysis showed that upon the knocking down of *prominin*-*like,* cell growth was delayed compared with the control cells. **d** Cellular ATP levels were measured using a bioluminescence assay, showing significant reductions of ATP levels in *prominin*-*like* RNAi-treated cells at day 2 and day 3 compared to control cells. The levels then recovered to almost normal at the third day after RNAi treatment (P value in the and third day was 0.759 and in the fourth day was 0.2492). **d**′ Measurement of mitochondrial ATP levels. Data showed significant reductions of mitochondrial ATP levels in *prominin*-*like* RNAi-treated cells at day 1–3 compared to control cells. The levels then gradually recovered to normal levels at the fourth day after RNAi treatment. **e** The rates of cellular ROS generation from *prominin*-*like* deficiency S2 cells and control cells. The result showed that the ROS production was upregulated in the short-term. **e**′ Measurement of mitochondrial ROS. Ratio of geometric mean intensity between levels of the ROS generation in the *prominin*-*like* knock down cells and control cells. Data showed significant increase of mitochondrial ROS levels in *prominin*-*like* RNAi-treated cells at day 1–2 compared to control cells. The levels then gradually recovered to normal levels at the fourth day after RNAi treatment. **f** Analysis of mitochondrial membrane potential (Δψm). Data are represented as mean ± SEM. *P < 0.05, **P < 0.01, ***P < 0.001

To analyze the biological function of Prominin-like, we employed double-stranded RNA (dsRNA)-mediated interference to down-regulate the expression of endogenous Prominin-like using S2 cells. The knockdown efficiencies were measured by both the transcription and protein levels of *prominin*-*like.* RT-PCR analysis showed that mRNA transcription levels of *prominin*-*like* in S2 cells treated with specific dsRNA fragments were dramatically reduced (by about 80%), compared with those of the control (negative control dsRNA treatment). This low *prominin*-*like* mRNA level persisted for 4 days after RNAi treatment (Fig. 3a). Western blot analysis indicated that the Prominin-like protein levels were also significantly decreased in dsRNA-treated S2 cells and that these low levels lasted for 4 days (Fig. 3b), which confirmed that the dsRNAi was functional.

We analyzed the cellular characteristics upon Prominin-like deprivation and found that the growth curve of Prominin-like deprived cells exhibited a delay of one day as compared with the control cells, followed by similar growth pace beyond (Fig. 3c). We have observed the morphology of the cells upon dsRNA treatment. There were no obvious differences between *prominin*-*like* knock down cells and the control cells under light microscopy, throughout day 1 to day 4 (Additional file 1: Figure S2). This probably indicated a transient effect of Prominin-like knockdown upon cell growth. Given the localization of Prominin-like in the mitochondria, we then tested whether ATP production would be affected upon *prominin*-*like* knockdown. The cellular ATP levels in the *prominin*-*like* dsRNAi treated S2 cells and the controls cells were measured using CellTiter-Glo^®^ Luminescent kit. Figure 3d shows that cellular ATP levels were significantly reduced by 12% at the first day and by 50% at the second day after RNAi treatment. Subsequently, the ATP levels were largely recovered at the 3^rd^ and 4^th^ days upon RNAi treatment, which is consistent with the state of the delayed cell growth rate. These results suggest that deficiency in Prominin-like leads to an acute ATP degeneration in the short term, along with a cell growth delay.

The mitochondrial ATP levels were detected using an ATP Bioluminescence Assay Kit. Significant reductions of mitochondrial ATP levels in *prominin*-*like* RNAi-treated cells were observed at day 1,2,3 (Fig. 3d′). The mitochondrial ATP levels then recovered to normal at day 4 after RNAi treatment. These results are similar to the cellular ATP levels as detected in *prominin*-*like* knockdown cells (Fig. 3d).

We also examined intracellular ROS levels in *prominin*-*like* knock down S2 cells using 2′,7′-dichlorodihydrofluorescein diacetate (DCFDA), a general oxidative stress indicator that could permeate the membrane and could be deacetylated then oxidized by ROS into 2′,7′-dichlorofluorescein (DCF) [17]. As shown in Fig. 3e, the levels of intracellular ROS generation had increased by approximately 50% on the first and second day after RNAi treatment, but were also largely recovered at the third day. The mitochondrial ROS production was examined using a MitoSOX assay. Compared to the controls, Mitochondrial ROS levels were increased by 21.9% at day 1 and by 35.2% at day 2 upon *prominin*-*like* knockdown and thereafter gradually recovered back to normal up to day 4 (Fig. 3e′). These data revealed that the alteration of both cellular and mitochondrial ATP and ROS production upon Prominin-like dsRNAi was consistent in terms of changes and duration, both displaying an effect that was both acute and transient.

### Knock down of *prominin*-*like* leads to mitochondrial dysfunction

The mitochondrial membrane potential (Δψm) is a key indicator of the integrality of mitochondrial structure and function [18]. The levels of Δψm were quantified by detecting the relative ratios of the FL_590_/FL_525_ geometric mean with JC-10 in both *prominin*-*like* knock down S2 cells and control cells. As shown in (Fig. 3f), the levels of *prominin*-*like* knock down S2 cells carryied only 77.4% (day 1), 70% (day 2) and 85% (day 3) of the those of the controls, while the Δψm levels had almost returned to that equivalent to those of the controls on day 4 after RNAi treatment.

**Fig. 4:**
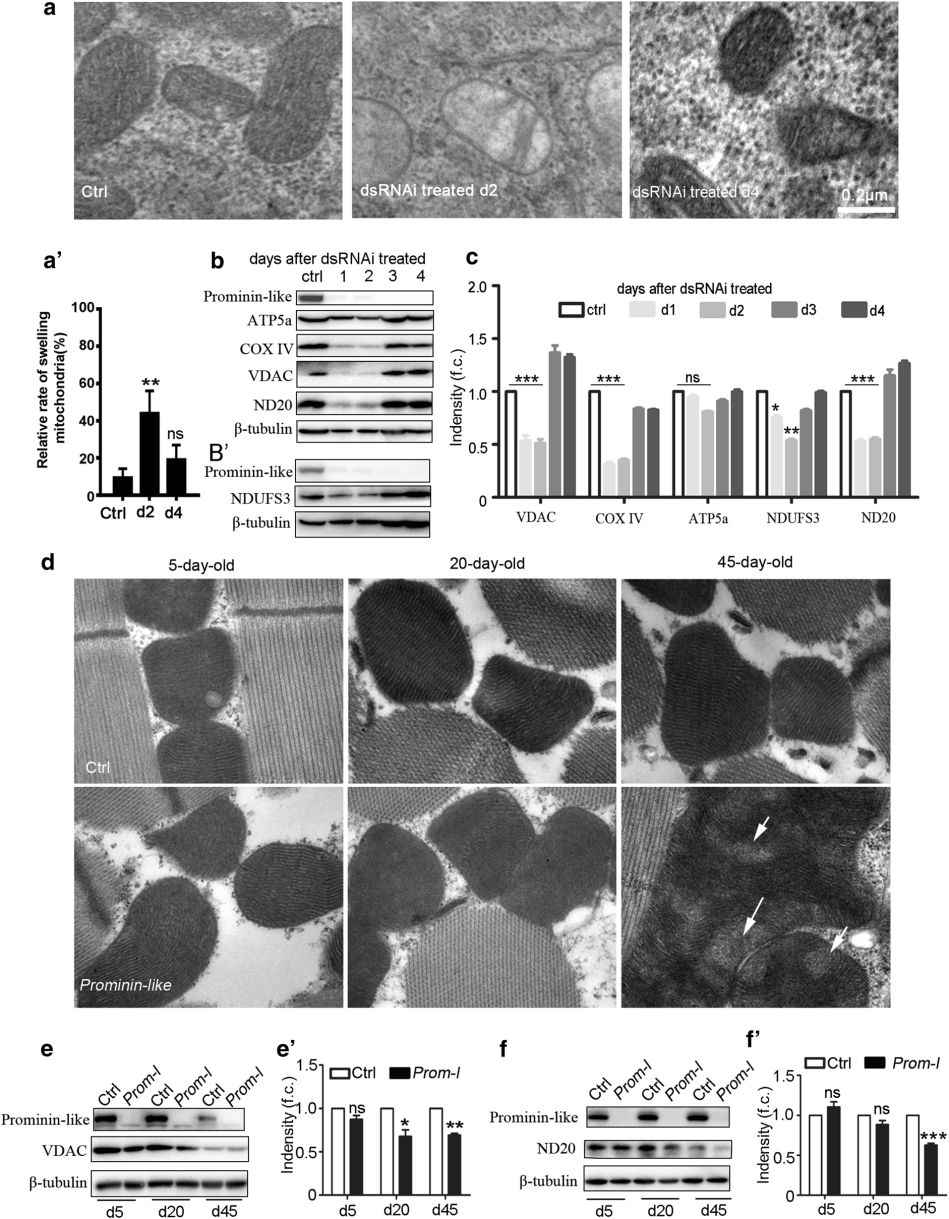
Knock down of *prominin-like* leads to mitochondrial dysfunction. **a** Transmission electron microscopy (TEM) images of S2 cells of Prominin-like knock down and control cells. Scale bars: 0.2 μm. **a**′ Statistical analysis of the rate of swelling mitochondria in *prominin*-*like* knock down cells (day2, n = 25; day 4, n = 26) and control cells (n = 21). **b**, **b**′ S2 cells were incubated with *prominin*-*like* dsRNA for 4 days. The protein expression levels of ATP5a, COX IV, NDUFS3, ND20 and VDAC were determined each day after RNAi treatment by Western blotting. β-tubulin expression was determined as a loading control under the same conditions. **c** Intensity measurement of the Western blot result in **b** by Image J software. **d** TEM images of *prominin*-*like* mutants and the control flies at various time points. The mitochondrial morphologies of 5-day-old and 20-day-old *prominin*-*like* mutants were similar to those of the control animals; while in 45-day-old mutants, the mitochondria showed obviously disordered mitochondrial cristae (arrows). **e** The protein expression levels of VDAC of *prominin*-*like* mutants and the control flies at vinous time points were determined by western blotting. **e**′ Intensity measurement of the western blot result in **e** by Image J software; Data showed that VDAC levels of *prominin*-*like* mutants were down-regulated in 45-day-old mutants not 5-day-old mutants. **b** The protein expression levels of ND20 of *prominin*-*like* mutants and the control flies at various time points were determined by western blotting. **f**′ Intensity measurement of the western blot result in **f** using Image J software. Data are represented as mean ± SEM. *P < 0.05, **P < 0.01

We next observed the mitochondrial morphology of the cells during the alteration period upon *prominin*-*like* dsRNAi treatment. The transmission electron microscopy (TEM) showed an obvious swelling of the mitochondrial cristae in Prominin-like down regulated S2 cells on the second day (Fig. 4a, middle). Statistical analysis showed that 44% mitochondria were swollen on the second day upon dsRNAi treatment, which represented obviously higher levels than the controls and day 4 cells (Fig. 4a′). This indicated that the mitochondrial dysfunction had occurred at that particular time. However, the mitochondrial morphology seemed to be normal in the control cells and in the treated cells on day 4 (Fig. 4a, right; a′).

We also examined whether the major mitochondrial proteins, that are responsible for mitochondrial ATP generation, were effected upon knockdown of Prominin-like in S2 cells. The expression levels of the VDAC located in the outer membrane [19], and the mitochondrial electron transport chain (ETC) complexes located in the inner membrane [20], were analyzed by Western blot. The results showed that the protein levels of VDAC as well as the ETC complexes subunits including NDUFS3,NADH dehydrogenase (ubiquinone), ND-20 and Cytochrome c oxidase subunit 4 (COX IV) were significantly reduced corresponding to the timing of decreased ATP levels after *prominin*-*like* dsRNA treatment (Fig. 4b, b′, c). The protein levels of ATP5a appeared to be unaffected (Fig. 4b, c).

As the decreased mitochondrial β-oxidation levels were detected in *prominin*-*like* mutants [14], we examined the mitochondrial status by TEM using the thoracic flight muscles of the *Prominin*-*like* mutant flies in terms of aging related alterations in mitochondrial morphology. Checked at day-5, day-20 and day-45, the mitochondria of thoracic flight muscles at 5-day-old and 20-day-old *prominin*-*like* mutants were similar to those of the control animals; while in 45-day-old mutants, the mitochondria showed obviously disordered mitochondrial cristae (Fig. 4d, arrows). We also checked the mitochondrial proteins including VDAC and ND20. Results were consistent with those of the mitochondrial morphology where both VDAC and ND20 protein levels were down-regulated in 45-day-old mutants, but not in 5-day-old mutants (Fig. 4e, f′). These data suggested that the knockdown of *prominin*-*like* might influence the mitochondrial electron transport chain and metabolite transporters, therefore effecting ATP production.

## Discussion

CD133 belongs to the 5-transmembrane glycoprotein prominin family consisting of three members, Prominin-1, 2, and 3 [21]. Studies using various cells, including hematopoietic stem cells [22], neural progenitor and epithelial cells [23], and hepatoma cells [24], have demonstrated that prominin-1/CD133 can be released with membrane vesicles through an endocytic–exocytic pathway. One recent study observed that when Src family tyrosine kinase activity was weak, non-phosphorylated CD133 combined with HDAC6 could be transported to the endosomes and was preferentially recruited to the pericentrosomal region via a dynein-based traffic system [25]. However, the role of CD133 in these cellular events remains unclear. Using *Drosophila* cells as a model system, here we report that the homolog of CD133, *Drosophila* Prominin-like, localizes on the cellular membranes and partially overlaps with mitochondria. ND20, as a subunit of mitochondrial respiratory complex I that oxidizes NADH [26], is confirmed here to interact with Prominin-like and together they maintain mitochondrial function related to ATP production.

Complex I is the largest complex of the respiratory chain consisting of at least 46 subunits. All except seven are encoded by the nuclear genome [27] and each play a unique role relating to the structure or activity of the complex. Complex I oxidizes NADH function to reduce ubiquinone and is coupled with proton translocation across the mitochondrial inner membrane [28]. One of the most common deficiencies of complex I involves defective oxidative phosphorylation and ROS generation [29]. In our experiments, decreased ATP production and increased ROS occurred upon the knockdown of *prominin*-*like*, which is an identical outcome to that of the knockdown of ND20 in S2 cells. Knockdown of *prominin*-*like* is also associated with changes in a number of mitochondrial proteins and alterations in mitochondrial morphology. In either knockdown of Prominin-like in S2 cells or in living Prominin-like mutant flies, an obvious decrease in ND20 is displayed. Such mitochondrial dysfunctions could be possibly triggered by the influence of Prominin-like deficiency on ND20, suggesting that Prominin-like is required for mitochondrial function.

In mammalian cells, it has also been reported that CD133 was able to translocate from the plasma membrane to the cytoplasm and in this way promote glucose uptake and ATP production under glucose starvation conditions [24]. CD133 also disrupts mitochondrial activity by inhibiting the endocytosis of transferrin [30], while bioenergetic stress caused by hypoxic and mitochondrial dysfunction induces a reversible up-regulation of CD133 expression [31]. The interaction of *Drosophila* Prominin-like and the ND20 that is active in the mitochondrial electron transport chain, suggests that its mammalian counterpart, CD133 might also be involved in the energy metabolism in similar mechanisms.

It is interesting to note that in S2 cells, the alterations of ATP and ROS, together with the expression levels of mitochondrial proteins including VDAC, NDUFS3, ND-20, and COX IV, lasted for 2 days after the down-regulation of *prominin*-*like* and then recovered to almost normal levels. This revealed that there exists a compensation pathway to rescue mitochondrial dysfunction caused by the knockdown of *prominin*-*like* and the consequent reduction of ND20 in S2 cells. In *prominin*-*like* mutant flies, we also found similar phenotypes related to mitochondrial dysfunctions, such as decreased mitochondrial β-oxidation levels and lower locomotion abilities [14]. In young *prominin*-*like* mutants, the mitochondrial morphology remains almost normal. However, mitochondrial dysfunction in older *prominin*-*like* mutants (Fig. 4d) still occurs and is probably due to accumulated defects beyond compensation occurring at early ages.

## Conclusion

In summary, we give the first demonstration that Prominin-like interacts with ND20 down-regulation of Prominin-like in *Drosophila* S2 cells leads to acute mitochondrial dysfunction. Conditional mitochondrial dysfunctions were also detected in aged *prominin*-*like* mutant flies. Our findings may provide some insight into the understanding of the similar function of CD133 in mammals.

## Methods

### Bioinformatics analysis tools

SMART (http://smart.embl-heidelberg.de/smart/set_mode.cgi?NORMAL=1) and TMHMM Server v2.0 (http://www.cbs.dtu.dk/services/TMHMM/) were used to detect predicted transmembrane domains in Prominin-like. The NetNGlyc 1.0 Server (http://www.cbs.dtu.dk/services/NetNGlyc/) was used to detect predicted N-Glycosylation sites. Clustal X 1.8 software was used for the multiple amino acid sequence alignments of various species, and the results were highlighted using DNAman software (version 6.0).

### Cell cultures and growth curve anaysis

The *Drosophila* S2 cell line was cultured at 25 °C without CO_2_ in 1× Schneider’s *Drosophila* medium (Gibco), supplemented with 10% heat-inactivated fetal bovine serum (FBS, Gibco). S2 cells grew as a loose semi-adherent monolayer, showing a doubling time of about 24 h. For cell growth curve analysis, the ratio of the viable cell numbers detected by the Trypan blue exclusion test against the cells at Time 0 (1× 10^6^/mL) was calculated. The assay was performed in triplicate and the average was calculated.

### Immunostaining and microscopy

The full length prominin-like cDNA was cloned into a pAC5.1 vector with a C-terminal Flag tag using primers:

- Forward: 5′-CCCCGGATCGGGGTACCTATGCCCACAACCGCCCAGGC-3′.
- Reverse: 5′-GCCGCCACTGTGCTGGATCTAATCCTGCTCGGAGGCAC-3′.
- MitoTracker^®^ Red CMXRos #9082

Cells were plated on poly-l-lysine-coated glass coverslips and transfection was carried out following the X-trmeGene HP (Roche) protocol. Two days after transfection, cells were collected and added MitoTracker^®^ Red (Cell Signaling) into the growth media at a concentration of 100 nM. After incubation for 30 min at 25 °C, cells were washed twice in 1× PBS and fixed in PBS with 4% paraformaldehyde for 20 min. Fixed cells were washed three times in PBS with 0.1% Triton X-100 (PBT) and blocked in PBT containing 3% BSA for 1 h at RT. Cells were then incubated with 1:500 monoclonal Rabbit-Flag antibody (Cell Signaling) at 4 °C overnight and washed again with PBT before incubating with 1:500 FITC-conjugated-rabbit IgG (Molecular Probes) for 2 h. DAPI (1 μg/mL, Sigma) was added for the last 20 min. After a final three washes in PBT, the coverslips were mounted in Vectorshield mounting medium. Images were taken using an Olympus FV1000 confocal microscope and processed using Adobe Photoshop. For transmission electron microscopy analysis, samples were processed according to the standard protocols and photographed using a Hitachi H-7650 transmission electron microscope.

### Mitochondrial fractionation

Cytosolic and mitochondrial fractions were isolated by differential centrifugation using a commercial kit (Mitochondria Isolation Kit for Tissue, 89801; Thermo Scientific). Both fractions were analyzed by Western blotting using the following antibodies: rabbit anti-Prominin-like (1:2000), mouse anti-ATP5A (1:1000, as mitochondrial loading control), and mouse anti-tubulin (1:1000, as cytosolic loading control).

### DsRNA synthesis and RNAi treatment

S2 cells were treated with double-stranded RNAi (dsRNA), as previously described [32]. dsCheck (http://dscheck.rnai.jp/), the web-based online software [33], was used to design target-specific dsRNA to avoid off-target silencing effects. A negative control (Nc) dsRNA was designed against a pBSSk (+) vector backbone. The T7 promoter sequence (TAATACGACTCACTATAGG) was added to the 5′terminus of all primers for dsRNA generation. The primer sequences (5′-3′) were as follows:

- PL-F, CCCAATTAAGAGATGGCGT,
- PL-R, GTCGAGTGCAGAGTTTTTATATG;
- Nc-F, TAAATTGTAAGCGTTAATATTTTG,
- Nc-R, AATTCGATATCAAGCTTATCGAT;

dsRNAs were produced using the MEGAscript RNAi Kit (Ambion) and were transfected into S2 cells following the standard protocol. Cells treated with Nc dsRNA served as wild-type controls for Prominin-like dsRNA treatment cells. The time after dsRNA treatment for 24 h at 25 °C, began to be counted as day 1, day 2, day 3 to day 4.

### Western Blotting

S2 cells were homogenized in a RIPA lysis buffer [5 mmol/L Tris–HCl, pH 8.0, 150 mmol/L NaCl, 1% (v/v) IGRPA CA-630, 0.5% (w/v) sodium deoxycholate and protease inhibitor cocktail (Roche)] and centrifuged at 12,000×*g* for 10 min at 4 °C to pellet nuclei and cell debris. The supernatant was collected and the protein concentration was assayed using a BCA protein assay kit (Beyotime). 20 μg of the protein samples were subjected to 12% sodium dodecyl sulfatepolyacrylamide gel electrophoresis (SDS-PAGE) and transferred to PVDF (polyvinylidene fluoride) membranes. The blotted membranes were immunoblotted with primary antibodies: rabbit anti-Prominin-like (1:2000), mouse anti-Tubulin (1:1000, DHSB E7), mouse anti-VDAC (1:1000, Abcam), mouse anti-Cytochrome C antibody (1:1000, Abcam 13575), mouse anti-ATP5a (1:1000, Abcam), mouse anti-COX IV (1:1000, Abcam), Rabbit anti-CG9172 (ND20) (1:500, Abgent), rabbit anti-HA (1:1000, Abcam), mouse anti-Flag (1:1000, Cell Signaling), rabbit anti-LAMP-1 (1:1000, Abcam). After secondary anti-rabbit horseradish peroxidase antibody labelling (Pierce; 1:5000), ChemiLucent™ ECL detection reagents (Millipore) was used to detect proteins and images were taken using the Chemiluminescence Imaging System (Clinx Science Instruments) as previously reported [34].

### Quantitative real-time PCR

Total RNA was isolated from S2 cells using a Trizol^®^ Reagent (Invitrogen). 1 μg RNA and First-Strand cDNA synthesis kit (Invitrogen) was used to produce single-stranded cDNA. Real-time PCR was performed with a Power SYBR Green PCR Master Mix (ABI) and an ABI 7900HT Fast real-time PCR system. Rp49 was used as an endogenous control. The following PCR primers (5′-3′) were used:

- *rp49*
- Forward: GCTAAGCTGTCGCACAAA
- Reverse: TCCGGTGGGCAGCATGTG
- *prominin*-*like*
- Forward: TCGATGTTGGCCACTGACGTG
- Reverse: TCCACTGTGGCAGCTACTTCA
- *VDAC*
- Forward: CAATGCCTCTGCCGTGCTT
- Reverse: CGAACTTGGTGTTGCTGGTG
- *COX IV*
- Forward: TGGGCGTTTCACTCCTCTT
- Reverse: GCCTTCTGGTGCTCCTCA
- *ATP5a*
- Forward: GTCGGTGTTGTGGTCTTCGG
- Reverse: ACGGTCCTTGGTGTTGATGG
- *CG9172*
- Forward: ACACCGAATGCGTCGATTTC
- Reverse: TGAGCAGATCGTCCAGTCTG

### Measurement of ATP levels

A CellTiter-Glo^®^ Luminescent Cell Viability Assay kit (Promega) was used to measure cellular according to the manufacturer’s instructions. 1 × 10^5^ S2 cells were seeded into a 96-well white opaque tissue culture plates and incubated with 50 μL assay reagent at 25 °C for 2 h. Then 50 μL Cell-Titer Glo Assay reagent was added to each well to induce cell lysis. After 10 min incubation at room temperature, the luminescence was measured by Syneregy H1 (Bio-Tek) as previously reported.

The ATP Bioluminescence Assay Kit HS II (Roche Applied Science) was used to measure mitochondrial ATP levels. S2 were incubated for 2 h in 1 mL recording solution (156 mmol/L NaCl, 3 mmol/L KCl, 2 mmol/L MgSO4, 1.25 mmol/L KH2PO4, 2 mmol/L CaCl2, 20 mmol/L HEPES, pH 7.35) with 5 mmol/L 2-deoxy-d-glucose (2-DG) plus 5 mmol/L pyruvate (oxidative ATP production). Cells were then lysed in lysis buffer (100 mmol/L Tris, 4 mmol/L EDTA, pH 7.75) and incubated for 5 min at 100 °C, the luminescence was measured by Syneregy H1 (Bio-Tek) as previously reported [35].

### Measurement of intracellular ROS

A DCFDA Cellular ROS Detection Assay Kit (Abcam) was used to measure intracellular reactive oxygen species (ROS) generation. Briefly, S2 cells were harvested by centrifugation at 100×*g* for 5 min and washed twice with PBS. Then cells were incubated with 1 mL PBS containing 10 μmol/L DCFH-DA (2,7-dichlorofluorescein diacetate) at 25 °C for 30 min. Cells were then washed again and resuspended in PBS with 50 μmol/L TBHB, followed by another 30 min culture at 37 °C. The emitted fluorescence from 1× 10^5^ cells, with or without TBHB stimulation, were determined using a Syneregy H1 (Bio-Tek) with an excitation wavelength of 485 nm and an emission wavelength of 535 nm.

### Measurement of mitochondrial ROS production

The levels of mitochondrial reactive oxygen species (ROS) generation were measured by using MitoSOX™ Red mitochondrial superoxide kit (M36008, Thermo Fisher). Experiments were conducted according to the manufacturer’s instructions. Briefly, S2 cells were harvested, washed twice with PBS, resuspended in 5 μM MitoSOX reagent working solution and then incubated at 25 °C for 20 min in darkness. The fluorescence intensities (excitation/emission [Ex/Em] = 510/580 nm) were measured on a multi-mode microplate reader (Synergy H1 Hybrid, BioTek).

### Mitochondrial membrane potential assay

The mitochondrial membrane potentials were analyzed using fluorescence detection (JC-10 Assay Kit, Abcam) according to the manufacturer’s instructions. In brief, the cells were plated onto 96-well, black-walled, clear-bottom plates and dyed with 50 μL of JC-10 solution. The fluorescence intensities (excitation/emission [Ex/Em] = 485/525 nm and Ex/Em = 540/590 nm) were measured on a multi-mode microplate reader (Synergy H1 Hybrid, BioTek). The ratio between aggregate (Em = 590 nm) and monomeric (Em = 525 nm) forms of JC-10 represents the change in the mitochondrial membrane potential. A high ratio indicates a high mitochondrial membrane potential [35].

### Yeast two-hybrid interaction assays

The assay was performed using the Matchmaker Gold Yeast Two-Hybrid system (Clontech), a GAL4-based yeast two-hybrid system, according to the instruction manual. In brief, the three large extracellular loops (PL1, PL2, PL3) and the intracellular C-terminal tail (PL4) cDNA were separately cloned into pGBKT7 DNA-BD vectors using EcoRI and BamHI sites as bait. The full-length cDNA of ND20 was cloned into pGADT7 AD vectors using EcoRI and BamHI sites as prey. The bait and prey clones were verified by sequencing. Then, bait vectors were transformed into a Y2H Gold yeast strain and the prey vectors were transformed into a Y187 yeast strain. We used an empty pGADT7 plasmid as a negative control (prey) for yeast two-hybrid analysis. All baits used in these experiments tested negative for auto-activation according to the manufacturer’s protocol. The haploid yeast strains harbouring each bait vector (Y2H Gold strain) and each prey vector (Y187 strain) were mated on 2× YPDA medium at 30 °C overnight. The diploid yeast cells that carried both bait and prey vectors were selected on both SD/− Leu/− Trp double dropout medium (DDO; for selection of bait and prey vectors), and SD/− Ade/− His/− Leu/− Trp/− X-α-Gal/AbA, Quadruple Dropout medium (QDO/X/A; for selection of both vectors and the interaction selection) with plates to score the protein–protein interaction by growth. After a 3-day incubation at 30 °C, images of the colonies on both plates were recorded.

### GST pull-down assay

The cDNA fragment encoding the peptide region of Prominin-like-1 (55–164aa) was cloned into pGEX-4T-1 vector and the products were transformed into *Escherichia coli* BL21 competent cells to produce a GST-PL1 fusion protein. GST or GST-PL1 proteins were separately expressed in *E. coli* BL21 and purified using Glutathione Sepharose 4B beads (GE Healthcare) according to the manufacturer’s instructions. In brief, BL21 cells expressing GST or GST-PL1 protein were treated with pull-down lysis buffer on ice for 30 min, followed by immobilization on an equilibrated glutathione agarose resin for 2 h at 4 °C. The resin was then washed five times with a wash solution (TBS: pull-down lysis buffer = 1:1).

The cDNA fragment encoding the peptide region of ND20, TSG101, HRB27C were cloned into pAHW vector by primers

- ND20-F: GCTGCTCATGGCGGACACCGGTCCATGCTGCGTTCGGCGATGAT
- ND20-R: CTTCACAAAGATCCTGCTAGCTTACTTCCTATACCACATCT
- TSG-F: GCTGCTCATGGCGGACACCGGTCCATGCCTGCGGTTGAGGAGAC
- TSG-R:CTTCACAAAGATCCTGCTAGCTTAGCCCGCTAGACCGGCCTT
- Hrb27cF: GCTGCTCATGGCGGACACCGGTCCATGGAGGAAGACGAGAGG
- Hrb27cR: CTTCACAAAGATCCTGCTAGCTTAGACAGCCTGCGAGGTTG

HA-ND20, HA-TSG101 and HA-HRB27C were generated in S2 cells through transfected cells. The S2 cell cultures were homogenized in a RIPA lysis buffer and the supernatants were added to the resin and incubated overnight at 4 °C. After washing five times, proteins were eluted with glutathione elution buffer. The eluted proteins were detected by Western blotting with an anti-HA antibody.

### Chemical cross-linking

For cross-linking studies, the reactions were performed at room temperature in Reaction Buffer (100 mmol/L HEPES, 50 mmol/L NaCl, pH 7.5). The membrane-impermeable cross-linker dithiobis-sulfosuccinimidyl-proprionate (DTSSP) (Thermo Scientific 21578) was added to 1 mM. After 30 min, cross-linking was quenched by the addition of a 37 mM final concentration of 1 mol/L Tris, pH 7.5 for 15 min.

### Co-immunoprecipitation (Co-IP)

S2 cells grown in six-well culture plates were transiently co-transfected with pAC5.1-Flag-PL1 and pAHW-HA-ND20 vectors by X-tremeGene HP (Roche Applied Science) or co-transfected with pAC5.1-Flag and pAHW-HA to serve as negative controls. At 48 h post-transfection, the cells were collected and washed with PBS then harvested with ice-cold modified RIPA lysis buffer [50 mmol/L Tris–HCl pH 8.0, 150 mmol/L NaCl, 1% (v/v) IGRPACA-630, 0.5% (w/v) sodium deoxycholate, complete protease inhibitor cocktail, PhosStop phosphatase inhibitor cocktail]. After centrifugation, one-tenth of the supernatant was used for input analysis, and the remainder of the supernatants were subjected to immunoprecipitation. Flag-tagged recombinant proteins were pre-cleared with Protein-G agarose beads (Roche Applied Science) for 3 h then captured with ~ 3 μg mouse anti-Flag antibody (Cell Signaling) on a rotating platform according to the manufacturer’s protocols. HA-tagged proteins were pre-cleared with Protein-A agarose beads (Roche Applied Science) then captured using ~ 3 μg rabbit anti-HA antibody (Abcam, ab9110). After rocking gently overnight at 4 °C, the beads were washed 5 times with ice-cold lysis buffer. Proteins binding to the beads were eluted with 2× SDS loading buffer at 95 °C for 10 min and subjected to SDS-PAGE followed by western blotting analysis with anti-HA or anti-FLAG antibodies.

## Supplementary information

**Additional file 1: Figure S1.** GST pull-down assays. GST or GST-PL-1 fusion protein generated by *E. coli BL21* cells was purified by glutathione agarose resin, followed by incubation of the resin with HA-TSG101(A) or HA-Hrb27C (B) protein expressed in S2 cells. After washing with PBS, the bound proteins were analyzed by Western blotting with anti-HA antibodies. Data showed that neither TSG101 nor HRB27C could be pulled down by Prominin-like. **Figure S2**. Images of S2 cells of prominin-like knock down and control cells. Scale bar: 50μm. Upon dsRNA treatment, the morphology of the treated cells seemed have no obvious differences between prominin-like knock down and control cells from day 1 to day 4, except for the cell density.

## Data Availability

Not applicable.

## Abbreviations

ATP5a: ATP-synthase complex V subunit alpha
Co-IP: co-immunoprecipitation
COX IV: Cytochrome c oxidase subunit 4
DCF: 2′,7′-dichlorofluorescein
DCFDA: 2′,7′-dichlorodihydrofluorescein diacetate
DDO: double dropout medium
Dlg: discs large
dsRNA: double-stranded RNA
DTSSP: dithiobis sulfosuccinimidyl proprionate
ETC: electron transport chain
GST: glutathione S-transferase
LAMP-1: lysosomal-associated membrane protein 1
ND20: NADH dehydrogenase 20 kDa subunit
PL: Prominin-like
QDO: quadruple dropout medium
S2: Schneider 2
TEM: transmission electron microscopy
VDAC: voltage-dependent anion channel
