# Supplementary material for: Drosophila Prominin-like, a homolog of CD133, interacts with ND20 to maintain mitochondrial function
Source: Cell Biosci. 2019 Dec 19;9:101. doi: 10.1186/s13578-019-0365-0 (PMC6923988; doi:10.1186/s13578-019-0365-0)
Supplement: Supplementary file 1 — Additional file 1: Figure S1. GST pull-down assays. GST or GST-PL-1 fusion protein generated by E. coli BL21 cells was purified by glutathione agarose resin, followed by incubation of the resin with HA-TSG101(A) or HA-Hrb27C (B) protein expressed in S2 cells. After washing with PBS, the bound proteins were analyzed by Western blotting with anti-HA antibodies. Data showed that neither TSG101 nor HRB27C could be pulled down by Prominin-like. Figure S2. Images of S2 cells of prominin-like knock down and control cells. Scale bar: 50μm. Upon dsRNA treatment, the morphology of the treated cells seemed have no obvious differences between prominin-like knock down and control cells from day 1 to day 4, except for the cell density. [file 13578_2019_365_MOESM1_ESM.docx]

**Additional file**


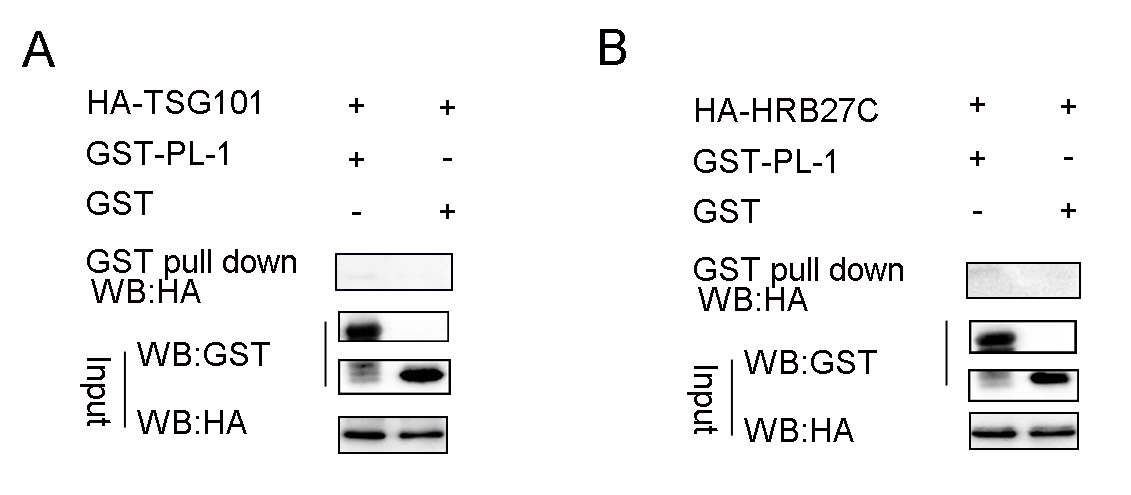


Figure S1. GST pull-down assays. GST or GST-PL-1 fusion protein generated by *E. Coli BL21* cells was purified by glutathione agarose resin, followed by incubation of the resin with HA-TSG101(A) or HA-Hrb27C (B) protein expressed in S2 cells. After washing with PBS, the bound proteins were analyzed by Western blotting with anti-HA antibodies. Data showed that neither TSG101 nor HRB27C could be pulled down by Prominin-like.


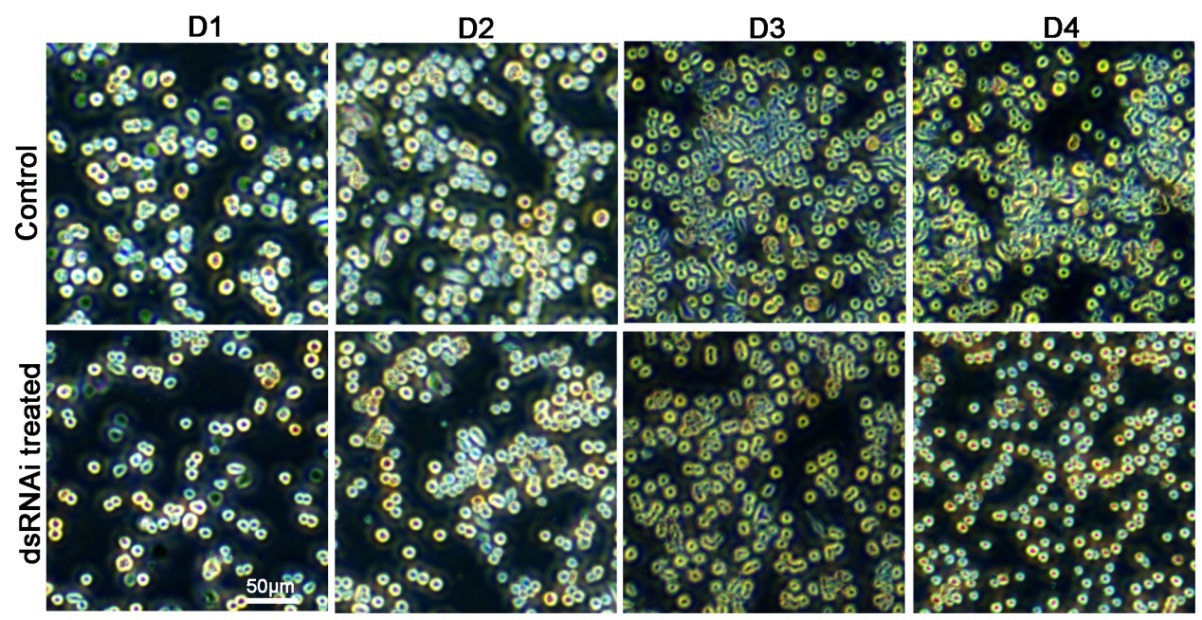


Figure S2. Images of S2 cells of prominin-like knock down and control cells. Scale bar: 50μm. Upon dsRNA treatment, the morphology of the treated cells seemed have no obvious differences between prominin-like knock down and control cells from day 1 to day 4, except for the cell density.
